# Target prediction by cascaded self-organizing maps for ligand de-orphaning and side-effect investigation

**DOI:** 10.1186/1758-2946-6-S1-P47

**Published:** 2014-03-11

**Authors:** Daniel Reker, Tiago Rodrigues, Petra Schneider, Gisbert Schneider

**Affiliations:** 1 Department of Chemistry and Applied Biosciences, Swiss Federal Institute of Technology (ETH), Zurich, 8093, Switzerland

## 

Computational chemogenomics approaches have emerged as a means to predict modulations of biomolecules by ligands. We implemented a method for the prediction of the macromolecular targets of small molecules combining state-of-the-art approaches that compare physicochemical properties and pharmacophoric features of query molecules with known drugs. Investigating similarity from multiple vantage points has been shown to increase the prediction accuracy in a retrospective evaluation. The method has been applied in multiple projects to “de-orphan” molecules with unknown main target and investigate potential side-effects of drug candidates. In a first application, the method identified a molecular scaffold as a potentially privileged structure of druglike compounds for chemoresistant tumor therapy [1]. In a second project, the tool revealed the potential of up to 5% of known bioactive substances to have unrecognized epigenetic effects by modulating histone deacetylase (HDAC) activity – thereby stressing the importance of probing for epigenetic effects in long-term drug toxicity studies [2].

## References

[B1] Reutlinger M Koch CP Reker D Todoroff N Schneider P Rodrigues T Schneider G Mol Inf 2013 32 133 138 10.1002/minf.201200141 PMC3743170 23956801

[B2] Lötsch J Schneider G Reker D Parnham MJ Schneider P Geisslinger G Doehring A Trends Mol Med 2013 in press 10.1016/j.molmed.2013.08.006 24054876

